# *Pseudonocardia* Symbionts of Fungus-Growing Ants and the Evolution of Defensive Secondary Metabolism

**DOI:** 10.3389/fmicb.2020.621041

**Published:** 2020-12-22

**Authors:** Sarah L. Goldstein, Jonathan L. Klassen

**Affiliations:** Department of Molecular and Cell Biology, University of Connecticut, Mansfield, CT, United States

**Keywords:** *Pseudonocardia*, attine ant mutualism, evolution, symbiosis, specialized (secondary) metabolite

## Abstract

Actinobacteria belonging to the genus *Pseudonocardia* have evolved a close relationship with multiple species of fungus-growing ants, where these bacteria produce diverse secondary metabolites that protect the ants and their fungal mutualists from disease. Recent research has charted the phylogenetic diversity of this symbiosis, revealing multiple instances where the ants and *Pseudonocardia* have formed stable relationships in which these bacteria are housed on specific regions of the ant’s cuticle. Parallel chemical and genomic analyses have also revealed that symbiotic *Pseudonocardia* produce diverse secondary metabolites with antifungal and antibacterial bioactivities, and highlighted the importance of plasmid recombination and horizontal gene transfer for maintaining these symbiotic traits. Here, we propose a multi-level model for the evolution of *Pseudonocardia* and their secondary metabolites that includes symbiont transmission within and between ant colonies, and the potentially independent movement and diversification of their secondary metabolite biosynthetic genes. Because of their well-studied ecology and experimental tractability, *Pseudonocardia* symbionts of fungus-growing ants are an especially useful model system to understand the evolution of secondary metabolites, and also comprise a significant source of novel antibiotic and antifungal agents.

## Introduction

Actinomycete bacteria form many beneficial symbioses with eukaryotes, where the host typically provides nutritional support and the actinomycetes provide chemical defense (Van Arnam et al., 2018). The best-studied of these are insect-actinomycete mutualisms, which are widespread and the source of many novel secondary metabolites with antibacterial and antifungal activity (Chevrette and Currie, 2019). Insect-associated *Streptomyces* inhibited clinically relevant microbes more effectively than soil-isolated *Streptomyces* (Chevrette et al., 2019), perhaps due to co-evolution between insects and microbes that has selected for defensive metabolites inhibiting pathogens but not their hosts (Clardy et al., 2009). The potential rise of antimicrobial resistance in these symbioses must have also been overcome by selection consistently replenishing and diversifying their defensive metabolites. However, few systems exist where such ecological and evolutionary dynamics have been dissected in detail.

## Fungus-Growing Ants: A Multipartite Mutualism

Fungus-growing (Attine) ants are one of the best-studied insect-microbe symbioses, encompassing > 250 described species from 17 genera (Schultz and Brady, 2008; Sosa-Calvo et al., 2018) that inhabit a geographic range stretching from the tip of Argentina to Long Island, New York, United States (Weber, 1972). Approximately 50–60 million years ago, these ants established a symbiotic relationship with a “cultivar” fungal symbiont that they farm in underground fungus gardens (Mueller et al., 1998). Fungus-growing ants provide fresh leaves (especially in the most-specialized leaf-cutting ants), grass clippings, fruits, berries, flowers, and insect frass to their fungal cultivar (De Fine Licht and Boomsma, 2010), which is the ants’ obligate food source. The cultivar relies on the ants for vertical propagation, and has lost its ability to reproduce sexually via spores (Weber, 1972). Virgin ant queens take a small piece of cultivar fungus from their native nests with them during their nuptial mating flights and use it establish their new colonies, propagating the fungal cultivar in a largely clonal fashion (Mueller et al., 1998). It was originally believed that no other fungi were present in ant fungus gardens due to the effects of antimicrobials that the ants secrete (Hölldobler and Wilson, 1990) and their extensive grooming behaviors (Currie and Stuart, 2001). However, Currie et al. (1999a) demonstrated the persistent presence of a specialized fungal parasite *Escovopsis* within ant fungus gardens that is highly pathogenic toward the cultivar fungus, and suggested that the fungus-growing symbiosis be expanded to include the ants, their cultivar, and the *Escovopsis* fungal pathogen as a coevolving tripartite symbiosis (Currie et al., 2003c). Future research will likely clarify the conditions under which *Escovopsis* acts as such a pathogen, and the impact of other pathogens in this symbiosis.

**FIGURE 1:**
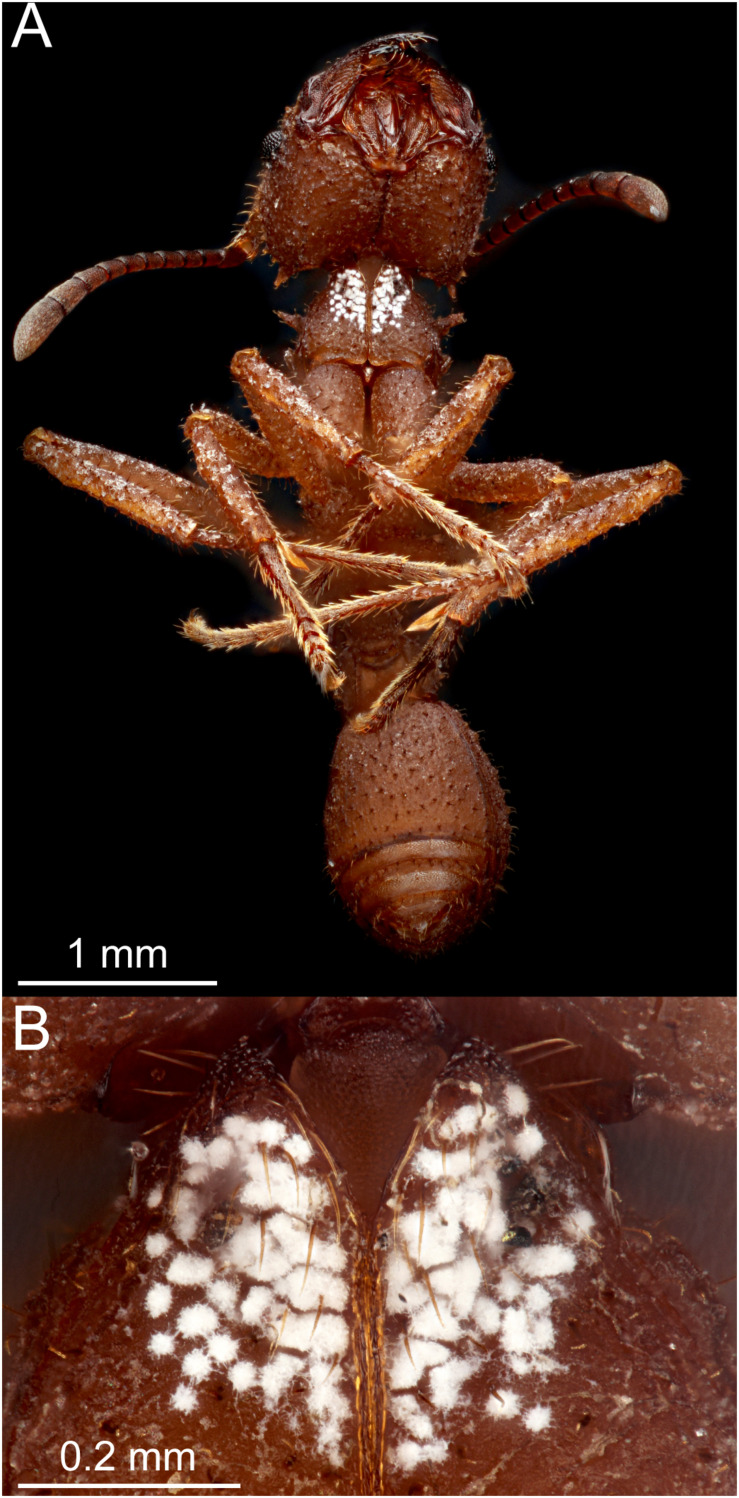
**(A)** Ventral view of an adult *Trachymyrmex septentrionalis* worker ant, showing the localization of *Pseudonocardia* (white patches) on the laterocervical plates that is typical of adult worker ants. **(B)** Enlargement of the laterocervical plates from **(A)**. Photo credit: Mark Smith, Macroscopic Solutions; used with permission.

Concurrent with the discovery of the fungal pathogen *Escovopsis*, Currie et al. (1999b) also established that an actinomycete bacterium comprises a fourth partner in the fungus-growing ant symbiosis. Many fungus-growing ant species have a region of their cuticle that is covered by a white or gray crust (Figure 1), which was initially described as a “waxy bloom” and dismissed as a cellular exudate (Weber, 1972). Upon closer inspection using scanning electron microscopy and targeted microbial isolations, this crust was subsequently determined to be a biofilm formed by the actinomycete *Pseudonocardia* (albeit initially misidentified as *Streptomyces*; Currie et al., 1999b, 2003b; Cafaro and Currie, 2005). These *Pseudonocardia* are housed in specialized structures on the ant cuticle that are connected to ant exocrine glands (Poulsen et al., 2003; Currie et al., 2006; Li et al., 2018), and their growth may be upregulated when an ant colony is under attack by *Escovopsis* (Currie et al., 2003a). *Pseudonocardia* symbionts can be parasitized by black yeast that compete with them for nutrients on the ant-cuticle, suppressing the growth of *Pseudonocardia* (Little and Currie, 2007). Such parasitism makes the fungus garden more susceptible to fungal infection, highlighting *Pseudonocardia*’s contribution to maintaining ant colony health (Little and Currie, 2008).

## *Pseudonocardia* as a Defensive Symbiont

Increased abundance of *Pseudonocardia* on ants in response to parasite infection underscores the predicted function of this bacterium in the system: to protect the fungal cultivar against *Escovopsis* (Currie et al., 1999b, 2003a). *Pseudonocardia* prevent *Escovopsis* infections of ant fungus gardens *in vivo* (Currie et al., 2003a; Little and Currie, 2008; Poulsen et al., 2010), and *Pseudonocardia* isolates consistently inhibit *Escovopsis* cultures *in vitro* (Currie et al., 1999b, 2003a; Schoenian et al., 2011; Meirelles et al., 2013; Sit et al., 2015; Dângelo et al., 2016). Some researchers have therefore suggested that these symbionts co-evolve with one another, locked in an arms race where *Pseudonocardia* and *Escovopsis* constantly evolve new mechanisms to gain an advantage over each other (Woolhouse et al., 2002). However, *Pseudonocardia* defenses against *Escovopsis* can vary (Poulsen et al., 2010), and some fungus-growing ants are not pathogenized by *Escovopsis* (Rodrigues et al., 2008), despite hosting *Pseudonocardia*. Further studies have shown that *Pseudonocardia* isolates have broad-spectrum activities against fungi other than just *Escovopsis* (Sen et al., 2009; Meirelles et al., 2013; Dângelo et al., 2016), suggesting that *Pseudonocardia*’s antimicrobials inhibit diverse pathogens in the fungus-growing ant symbiosis.

*Pseudonocardia* strains can also inhibit entomopathogens that infect the ants (Sen et al., 2009; Mattoso et al., 2012), to which ants are inevitably exposed to as they excavate tunnels, tend to brood, or forage for plant matter (Hughes et al., 2004, 2009). Although the ants themselves possesses an innate immune system that can defend against pathogens (Gillespie et al., 1997), and engage in allogrooming to reduce the potential for infection (Walker and Hughes, 2009), *Pseudonocardia* may add further protection against ant pathogens (de Souza et al., 2013). *Pseudonocardia* abundance peaks at 10–15 days post-eclosion before declining (Poulsen et al., 2003), and so *Pseudonocardia* may therefore particularly confer protection to young workers by giving their immune systems time to develop and recognize entomopathogens, in addition to protecting the fungal cultivar against *Escovopsis* (de Souza et al., 2013).

## *Pseudonocardia* Symbiont Transmission and Specificity

*Pseudonocardia* are thought to typically be transmitted vertically, similar to the fungal cultivar. *Pseudonocardia* symbionts were detected on foundress ant queens, but not on male alates, during their nuptial flights (Currie et al., 1999b). *Pseudonocardia* were also identified on virgin queens within their native nests, but not on males (Currie et al., 1999b), suggesting that founding queens need to maintain *Pseudonocardia* to successfully establish new colonies. Once a colony is established, *Pseudonocardia* are transmitted vertically to new workers within 2 h of eclosing via contact with an older worker ant that has an established *Pseudonocardia* biofilm, after which time vertical transmission is drastically reduced (Marsh et al., 2014). Vertical transmission of *Pseudonocardia*, therefore occurs both within and between ant colonies.

Phylogenetic studies have also revealed an evolutionary history of ant-associated *Pseudonocardia* that is largely, but not exclusively, consistent with vertical transmission. Most fungus-growing ant colonies maintain a single strain of *Pseudonocardia* (Poulsen et al., 2005; Andersen et al., 2013), and this specificity can be maintained in the lab for at least 10 years (Andersen et al., 2013). Consistent with the dominance of vertical transmission, Cafaro et al. (2011) observed significant, but not absolute, patterns of specificity between lineages of *Pseudonocardia* and their ant host genera using a multi-locus gene phylogeny. Subsequent studies indicated that the *Pseudonocardia*-ant symbiosis has been gained and lost multiple times during ant evolution (Li et al., 2018), the most notable of these being the loss of *Pseudonocardia* in the highly derived *Atta* leaf-cutting ants (but see Marsh et al., 2013). *Pseudonocardia* symbionts of *Apterostigma dentigerum* ants have population structures that are consistent with vertical transmission between their dispersal-limited hosts (Caldera and Currie, 2012; Mcdonald et al., 2019), but similar population structures were not detected for *Pseudonocardia* symbionts of *Trachymyrmex septentrionalis* ants using methods with lower phylogenetic resolution (Mikheyev et al., 2008). Ants can recognize their native *Pseudonocardia* symbiont (Zhang et al., 2007; Poulsen et al., 2011), and experimental symbiont swaps decrease symbiont abundance and ant grooming behavior, thereby allowing increased pathogen infection (Armitage et al., 2011; Andersen et al., 2015). These results show that *Pseudonocardia* can be adapted to their specific ant hosts and vice versa, as expected from a predominantly vertical mode of transmission, although symbiont replacement remains possible.

Like many microbial symbionts (Garcia and Gerardo, 2014), the fitness benefits that *Pseudonocardia* gain from their relationship with fungus-growing ants remains unclear. Although exocrine gland secretions have been speculated to feed *Pseudonocardia* symbionts (Currie et al., 2006), this has not been demonstrated unequivocally. The vertical transfer of *Pseudonocardia* between ant generations implies fitness benefits that are received by these bacteria (otherwise the relationship would be expected to break down). However, *Pseudonocardia* presence varies between related ant species (Fernández-Marín et al., 2013) and genera (Li et al., 2018), indicating that the benefits of this relationship change over time. Further research is warranted to determine the conditions under which selection favors *Pseudonocardia* and/or their ant hosts, and how potential conflicts between these partners are resolved, which will define when and if *Pseudonocardia* functions as an ant mutualist, commensal, or parasite.

The predicted function of *Pseudonocardia* as a defensive symbiont provides an evolutionary incentive for ant colonies to maintain effective *Pseudonocardia* strains. There is a fitness cost for an ant to swap symbionts if a less effective strain replaces a more effective one (Sachs et al., 2011). However, strict maintenance and vertical transmission of clonal symbionts can lead to other potential problems, such as Muller’s ratchet, which predicts that a symbiont is ultimately doomed to extinction due to the accumulation of deleterious mutations in the absence of recombination or symbiont replacement (Bennett and Moran, 2015). Considering that *Pseudonocardia* symbionts of fungus-growing ants were observed in a piece of 15 million year old amber (Li et al., 2018), it is likely the ant-*Pseudonocardia* symbiosis has been conserved over long evolutionary timescales, despite the predominantly vertical transmission of clonal symbiont populations.

## Challenging *Pseudonocardia* Specificity and Clonality

How then does this ant-actinomycete symbiosis maintain enough diversity to avoid extinction or the loss of their defensive function? One hypothesis is that actinomycetes other than *Pseudonocardia* are also maintained as defensive symbionts of ants. Several studies have isolated such actinomycetes from fungus-growing ants and showed that they inhibit fungal pathogens *in vitro* (Kost et al., 2007; Mueller et al., 2008; Haeder et al., 2009; Sen et al., 2009; Barke et al., 2010; Dângelo et al., 2016). Schoenian et al. (2011) also found compounds known to be produced by *Streptomyces* strains on the cuticle of *Acromyrmex* ants, concluding that those actinomycetes were therefore ant symbionts. However, these studies have limitations that constrain their ability to unambiguously assign a symbiotic relationship or defensive function to these actinomycetes (Klassen, 2014, 2018, 2020). First, they typically sample few ant colonies, in contrast to the systematic sampling of *Pseudonocardia* that shows its widespread relationship with fungus-growing ants (Cafaro et al., 2011; Li et al., 2018). Such limited sampling cannot differentiate persistent symbionts from more transient microbial contaminants. Second, the widely used culture-based techniques are largely qualitative and can misrepresent the dominant taxa in samples (Andersen et al., 2013), instead leading to a focus on low-abundance microbes due to enrichment biases. Third, samples taken from whole ants instead of the specific locations where *Pseudonocardia* are known to localize (Figure 1) can introduce contaminants that mask the dominance of *Pseudonocardia* and its products in their more specific niche (Andersen et al., 2013; Gemperline et al., 2017). Thus, although it may be true that other actinomycetes occur in the fungus-growing ant symbiosis and produce secondary metabolites, additional evidence is required to confirm their functional role as fungus-growing ant symbionts and to rule out alternative interpretations such as transient contamination of ant colonies (Klassen, 2014, 2018, 2020).

The clonality of *Pseudonocardia* symbionts within individual ant colonies has also been challenged. Culture-dependent and -independent 16S rRNA gene amplicon sequencing of *T. septentrionalis*-associated *Pseudonocardia* found an average of 2.9 strains of *Pseudonocardia* per ant (Ishak et al., 2011). However, this study sampled whole ants and ant sections instead of specifically targeting the propleural plates where *Pseudonocardia* is localized, perhaps including transient bacteria from within the ant and elsewhere on the cuticle. These criticisms also apply to similar studies (e.g., Sen et al., 2009), including those that sampled *Pseudonocardia* from ant fungus gardens instead of from on the ants themselves (e.g., Mueller et al., 2008). In contrast, laterocervical plates dissected from *Acromyrmex echinatior* ants with the remaining internal soft tissue removed prior to 454 16S rRNA gene pyrosequencing hosted single *Pseudonocardia* strains in 25 of 26 ants sampled (Andersen et al., 2013), consistent with the prevalence of clonal *Pseudonocardia* populations in most, but not all, fungus-growing ant colonies. Finally, it is important to note that these and related studies investigating the clonality and transmission of *Pseudonocardia* strains (e.g., Mueller et al., 2010) have relied on the partial sequencing of housekeeping genes that contain limited phylogenetic information and that are often superseded by the higher resolution provided by whole genome sequencing, which is able to more precisely resolve species and population-level differences (e.g., Mcdonald et al., 2019).

## Competition May Drive Horizontal Gene Transfer

Despite the issues described above, the presence of other actinomycetes in association with fungus-growing ants should not be discounted. *Pseudonocardia* isolates may be maintained and vertically propagated in this symbiosis while also acquiring genetic diversity, particularly secondary metabolite biosynthetic gene clusters (BGCs), via genetic exchange with other environmental actinomycetes. This strategy would allow *Pseudonocardia* to avoid the consequences of strict vertical transmission, such as Muller’s ratchet, and to increase their fitness by acquiring BGCs from other actinomycetes to overcome pathogen resistance. The ability to acquire BGCs may represent a preadaptation that makes *Pseudonocardia* an especially successful symbiotic partner (Toft and Andersson, 2010). Horizontal acquisition of defensive genes may also provide *Pseudonocardia* with the ability to compete against other strains that seek to colonize the ant host (Sachs et al., 2011). This ability to inhibit other actinomycetes may even have given rise to the vertical propagation of specific *Pseudonocardia* lineages, allowing what may have initially began as a parasitic relationship to transition to a mutualism (Sachs et al., 2011). Other theoretical models have suggested that such competition between actinomyces may actively select for *Pseudonocardia* that produce high levels of bioactive compounds on the ants (Scheuring and Yu, 2012), although it should be noted that the antibacterial compounds deployed for competition between bacteria are likely to differ from those that mediate antifungal defense.

Native *Pseudonocardia* strains inhibit the growth of other *Pseudonocardia* that may seek to take over the ant cuticle. Resident *Pseudonocardia* strains inhibited ∼60% of tested intruder strains, and most strongly inhibited intruders that were genetically distant from the resident strain, including strains from other fungus-growing ant species and non-ant environments (Poulsen et al., 2007). This pattern may result from genetically related *Pseudonocardia* possessing similar BGCs, and therefore similar resistance genes that are often genetically linked to these BGCs. Two *Pseudonocardia* isolates, BCI1 and BCI2, were isolated from *A. dentigerum* ants collected on Barro Colorado Island (BCI), located in the middle of the Panama Canal. These strains shared 100% identical 16S rRNA genes and >98% average nucleotide identity between their chromosomes (Van Arnam et al., 2015). However, only strain BCI2 inhibited all other tested actinomycete strains due its unique acquisition of a BGC that encoded for an analog of the antimicrobial metabolite rebeccamycin on a plasmid that was otherwise >96% conserved in strain BCI1. The presence of this novel plasmid-encoded BGC suggests that strain BCI2 acquired these genes horizontally from environmental actinomycetes. Similarly, Chang et al. (2020) recently isolated thiopeptide GE37468 from *Trachymyrmex septentrionalis* ants, whose BGC was closely related to that of the non-symbiotic *Streptomyces* strain ATCC 55365. It is therefore, likely that *Pseudonocardia* symbionts acquire BGCs from environmental actinomycetes.

## *Psseudonocardia* as a Resource for Novel Metabolite Discovery

Both known and unknown antimicrobials have been identified from *Pseudonocardia* symbionts of fungus-growing ants and the antibiotics GE37468 (Chang et al., 2020), X-14881 E, and 6-deoxy-9-O-methylrabelomycin (Carr et al., 2012). Novel metabolites discovered from *Pseudonocardia* symbionts include the antibiotics pseudonocardone A, B, and C (Carr et al., 2012), and 9-methoxyrebeccamycin (Van Arnam et al., 2015), the depsipeptide natural products dentigerumycin (Oh et al., 2009) and gerumycin A, B, and C (Sit et al., 2015), nystatin-like antifungals (Barke et al., 2010; Seipke et al., 2012; Holmes et al., 2016), and the atypical antifungal polyene selvamicin (Van Arnam et al., 2016). The variable genetic contexts in which the BGCs encoding for these metabolites occur is striking. The gerumycin BGC is encoded chromosomally in one *Pseudonocardia* strain but on the plasmid of another (Sit et al., 2015); the same is true for selvamicin (Van Arnam et al., 2016). This suggests that *Pseudonocardia* strains may horizontally acquire BGCs first on their plasmids, and then later move them to their chromosome. Alternatively, BGCs could move from the chromosome to plasmid(s), with either mechanism generating high levels of BGC diversity on plasmids that might be a fruitful target for the discovery of novel metabolites (Sit et al., 2015; Ruzzini and Clardy, 2016).

*Pseudonocardia* symbionts also vary in their BGC composition over local geographic scales. BGC composition varied between strains sampled across a 20 km transect in Panama (Mcdonald et al., 2019). Of the 27 BGC families identified from these *Pseudonocardia* symbionts, 7 occurred only on BCI. *Pseudonocardia* symbiont strains obtained from BCI also displayed local adaptation to the *Escovopsis* strains that were endemic to that location (Caldera et al., 2019), suggesting that this may be a hotspot for evolving antifungal bioactivities. Other such hotspots likely exist throughout fungus-growing ant biodiversity and could also be targeted for the discovery of novel metabolites.

## Conclusion

**FIGURE 2:**
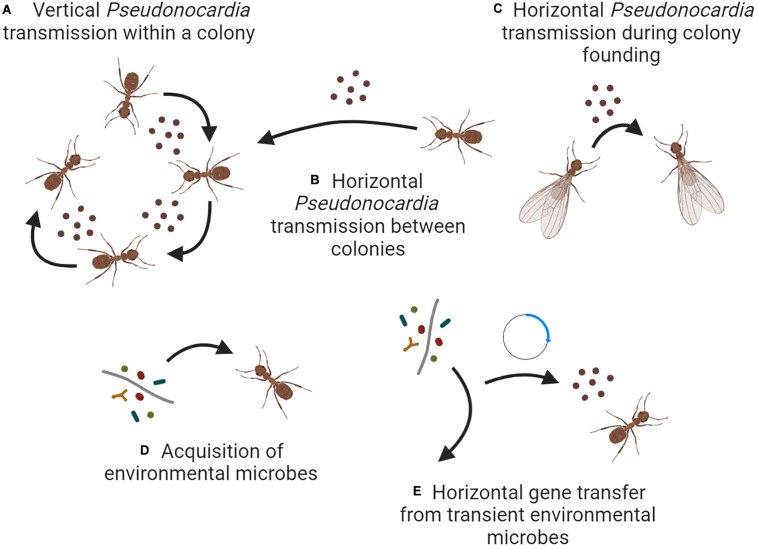
Ecological and evolutionary mechanisms that may govern the diversity of *Pseudonocardia* fungus-growing ant symbionts. Scenarios **(A–C)** all describe transmission involving other *Pseudonocardia* symbionts, either vertically within an established ant colony **(A)**, horizontally between established colonies **(B)**, or during colony founding **(C)**. Scenario **(D)** describes the acquisition of new symbionts from the external environment, and scenario **(E)** describes the horizontal transfer of genes from those environmental microbes without acquisition of the microbes themselves. Note that the experimental evidence supporting each scenario varies. Figure created with BioRender.com.

Having been initially established multiple times (Li et al., 2018), the fungus-growing ant-*Pseudonocardia* symbiosis now evolves simultaneously on multiple organizational levels. First, *Pseudonocardia* strains are occasionally transferred horizontally between colonies, despite the predominance of vertical transmission between ants and ant colonies (Figures 2A,B). Although data is lacking, such transfer may most likely occur during colony founding when the source populations of *Pseudonocardia* are small and therefore, more prone to stochastic variation (Figure 2C; cf. Poulsen et al., 2009). Transfer between colonies may also be facilitated by antibiotics that allow invasion that overcomes native *Pseudonocardia* strains (Poulsen et al., 2007; Van Arnam et al., 2015). Second, niche-defining genes such as secondary metabolite BGCs may be horizontally transferred to *Pseudonocardia* from other microbes that pass through its ant-associated niche, and such transfer likely involves plasmids as a prominent mechanism of genome plasticity (Figures 2D,E; Van Arnam et al., 2015). Together, these mechanisms allow *Pseudonocardia* symbionts to avoid Mueller’s ratchet and maintain their effectiveness as defensive mutualists of fungus-growing ants.

## Author Contributions

SG and JK conceived, wrote, and revised this manuscript. Both authors contributed to the article and approved the submitted version.

## Conflict of Interest

The authors declare that the research was conducted in the absence of any commercial or financial relationships that could be construed as a potential conflict of interest.
